# GmMPK6 Positively Regulates Salt Tolerance through Induction of *GmRbohI1* in Soybean

**DOI:** 10.3390/antiox12030601

**Published:** 2023-02-28

**Authors:** Seungmin Son, Jitae Kim, Chung Sun An, Song Lim Kim, Hyoungseok Lee, Jong Hee Im

**Affiliations:** 1 School of Biological Sciences, College of Natural Sciences, Seoul National University, Seoul 08826, Republic of Korea; 2 National Institute of Agricultural Sciences, Rural Development Administration, Jeonju 54874, Republic of Korea; 3 Bioenergy Research Center, Chonnam National University, Gwangju 61186, Republic of Korea; 4 Division of Life Sciences, Korea Polar Research Institute, Incheon 21990, Republic of Korea; 5 Department of Horticulture, Michigan State University, East Lansing, MI 48824, USA; 6 Great Lakes Bioenergy Research Center, Michigan State University, East Lansing, MI 48824, USA

**Keywords:** mitogen-activated protein kinase, reactive oxygen species, respiratory burst oxidase homolog, salt stress, soybean

## Abstract

Salt stress is a critical environmental stress that impairs plant growth and development, especially in crop productivity; therefore, understanding the salt response in plants is the basis for their development of salt tolerance. Under salinity, soybean mitogen-activated protein kinase 6 (GmMPK6) is activated and positively regulates reactive oxygen species (ROS) generation. However, it is not yet elucidated how GmMPK6 regulates ROS generation and its role in salt tolerance. Here, we show that GmMPK6, solely activated in NaCl treatment, and gene expression of *GmRbohI1* was not only reduced by MPK inhibitor SB202190 in NaCl treatment, but also increased in a GMKK1-expressing protoplast. Furthermore, SB202190 and the NADPH-oxidase inhibitor, diphenyleneiodonium chloride, increased susceptibility to salt stress. The expression of *GmRD19A* was induced by NaCl treatment, but this expression was compromised by SB202190. Consequently, we revealed that GmMPK6 induces ROS generation through the transcriptional regulation of *GmRbohI1* and increases salt tolerance in soybean.

## 1. Introduction

Salt is one of the most destructive environmental stresses and damages the yield and quality of crops all over the world [1]. Environment changes, such as low precipitation and high surface evaporation, are the main causes of soil salinization, and more than 50% of arable land is expected to be salinized within 30 years [2,3]. Therefore, salinized soil caused by climate change is expected to have a serious adverse effect on crop nutrient security. However, understanding the defense mechanism conferring salinity tolerance is limited in many crop plants, including soybean (*Glycine max*), which provides an important source of food, protein, and oil.

A high salt concentration causes ionic and osmotic imbalances, which can induce oxidative stress due to the accumulation of reactive oxygen species (ROS) in plant cells [4,5,6]. ROS, including radicals (e.g., O_2_^•−^ and OH^•^) and non-radicals (e.g., H_2_O_2_ and ^1^O_2_), are constantly generated by aerobic metabolism in various plant cellular compartments (e.g., chloroplast, mitochondria, peroxisomes, apoplast, and endoplasmic reticulum) [7]. Although ROS can cause oxidative stress, they also play a role as important signaling molecules, regulating various signaling pathways related to biotic/abiotic stresses in plant cells [8,9,10]. Therefore, the equilibrium between ROS generation and detoxification is strictly regulated by antioxidant defense systems under stress conditions [11,12].

Under harsh environmental conditions, including salinity stress, metabolic pathways (metabolic ROS) and stress signaling pathways (signaling ROS) mainly produce ROS in plant cells [13]. NADPH-oxidase called respiratory burst oxidase homolog (Rboh) localizes on the plasma membrane and plays a key role in the generation of signaling ROS in plants [14,15]. Many studies show that Rboh is involved in and enhances salt tolerance in various plants. ROS generation by AtRbohD and AtRbohF was necessary for enhancing salt tolerance in *Arabidopsis thaliana* [16,17,18]. The induction of *GmRbohI* homologous genes (*GmRbohB-1* and *GmRbohB-2*) by the salt-induced NAC1 (GmSIN1) transcription factor resulted in a rapid accumulation of ROS and activated an initial salt stress signal conferring salt tolerance in soybean [19]. The NtbHLH123 transcription factor also enhanced the salt tolerance of *Nicotiana tabacum* through the induction of the *NtRboh* gene and ROS production [20]. Moreover, it was revealed that K^+^ homeostasis modulated by OsRbohA-mediated H_2_O_2_ accumulation is important for salt tolerance in rice [21]. However, knowledge of the regulation mechanisms of Rboh in salt stress response is still limited in plants.

Mitogen-activated protein kinase (MPK) plays an important role in various cellular signaling pathways [22] and increases ROS generation in various plants. For example, INF1 dependently activates NTF4, and NTF6 activates NbRboh in tobacco [23]. In Arabidopsis, the MKK5-MPK6 module regulates superoxide dismutase in salt stress [24]. ROS also activates MPK activity. Arabidopsis ANP1 initiated H_2_O_2_-dependent AtMPK3/6 activity and induces stress-responsible genes [25]. ABA dependently activated 46 kDa MPK, which is also activated by H_2_O_2_ in maize [26]. The OXI1-MPK6 module is activated by ROS, and MPK6 activates NADPH-oxidase [22]. These data suggest that ROS-MPK has a feedback mechanism, but it is not clear which one comes first.

In soybean, salt stress activates GmMPK6 (GMK1) through phosphatidic acid and hydrogen peroxide (H_2_O_2_) [27,28]. Moreover, the nuclear translocation of GmMPK6 by salt stress is mediated by H_2_O_2_ [29], and MPK inhibitor SB202190 reduces GmMPK6 activity and ROS generation in salt stress [28], suggesting that GmMPK6 activity is related to ROS generation under salt stress. However, the molecular function of GmMPK6-dependent ROS generation and how GmMPK6 regulates ROS generation during salt stress are elusive. Here, we show that a GmMPK6-mediated signaling process positively regulates the transcriptional activation of *GmRbohI1* to generate cellular ROS, which can result in enhanced salt tolerance in soybean. 

## 2. Materials and Methods

### 2.1. Plant Growth and Treatments

Soybean (*Glycine max*) seeds were germinated in wet paper towels in dark condition for 3 days, and they were then grown for 4 days in a Light-Safe 50 mL conical tube (Stellar Scientific, Owings Mills, MD, USA) with half hypocotyl submerged in B & D solution [30]. For inhibitor treatment, the seedlings were pretreated with inhibitors for 60 min to roots and hypocotyls and treated with the NaCl solution for the desired time; they were then used for gene expression analysis and ROS generation analysis. Four-day-old seedlings were used for seedling phenotype analysis in NaCl and pharmaceutical co-treatment. Ten-day-old seedlings grown in B & D solution were also used for phenotype and chlorophyll content analysis with 0.15 M NaCl and pharmacological co-treatment (n = 8).

### 2.2. RT-qPCR

After NaCl treatment on soybean seedlings with or without inhibitors treatment, total RNA was isolated from the seedlings using RNeasy Plant Mini Kit (Qiagen, Germantown, MD, USA). First-strand cDNA was synthesized from 200 ng total RNA for each sample using SuperScript™ II Reverse Transcriptase (Invitrogen, Waltham, MA, USA). qPCR was carried out with specific primers (Table S1) on a 7500 Real-Time PCR System (Applied Biosystems, Waltham, MA, USA) using a comparative Ct method [31] with Fast SYBR™ Green Master Mix (Applied Biosystems, Waltham, MA, USA). Because salt stress affect various gene expressions [32], we tested *GmRbohI1* expression with three different reference genes, *GmActin*, *GmTubulin*, and *GmUBQ*. Because the expression of *GmRbohI1* normalized to the three reference genes showed the same gene expression pattern (Figure S1), we used *GmActin* as a reference gene in subsequent experiments.

### 2.3. ROS Measurement

Seven-day-old soybean seedlings were pre-treated with or without 200 μM cycloheximide (CHX), 30 μM SB202190, or 50 μM NADPH-oxidase inhibitor, Diphenyleneiodonium chloride (DPI) and simultaneously treated with 0.3 M NaCl and 0.5 mM 2,3-bis-(2-methoxy-4-nitro-5-sulfophenyl)-2H-tetrazolium-5-carboxanilide (XTT; Sigma, St. Louis, MO, USA) for the desired time, and then 200 μL of the NaCl-XTT solution was used for measuring XTT reduction using a spectrophotometer at an absorbance wavelength of 470 nm.

### 2.4. Protoplast Isolation and PEG Transfection

The protoplast isolation method used in this study was previously described in [28]. Briefly, the roots and hypocotyls of 7-day-old soybean seedlings that were grown in dark conditions were cut to a size of 1 mm and transferred to an enzyme solution (1% *w*/*v* of cellulase RS (YAKULT, Tokyo, Japan) and macerozyme R-10 (MB cell, Korea), 0.4 M mannitol, 20 mM KCl and MES, 10 mM CaCl_2_, and 0.1% BSA). After the solution was incubated at room temperature for 3 h, the solution was filtered through an 80 μm nylon mesh, centrifuged at 200× g for 3 min, and resuspended in W5 solution (150 mM NaCl, 125 mM CaCl_2_, 5 mM KCl, and MES; pH 5.7). The cells were precipitated by gravity, and W5 solution was removed, and the cells were resuspended in MMG solution (0.4 M mannitol, 15 mM MgCl_2_, and 4 mM MES, pH 5.7). PEG transfection of GMKK1 construct was performed as previously described [28,33].

### 2.5. Chlorophyll Content Analysis

Total chlorophyll content was analyzed as previously described [34]. Briefly, pigments of equal fresh weights of soybean leaves were extracted with 80% acetone solution. The content of total chlorophyll was measured using a UV/VIS spectrophotometer (Thermo Fisher Scientific, Waltham, MA, USA) and calculated as previously described [35].

### 2.6. Immunodepletion and In-Gel Kinase Assay

Seven-day-old soybean seedlings were treated with 0.3 M NaCl for 5 min and extracted total protein with protein extraction buffer (50 mM Tris-HCl (pH 7.4), 1% NP-40, 150 mM NaCl, 1 mM EDTA, 1 mM PMSF, 1 mM Na_3_VO4, 1 mM NaF, and 1 mg/mL aprotinin, leupeptin, and pepstatin). For immunodepletion assay, anti-GmMPK6 antibody [28] was added to the protein and incubated for 2 h at 4 °C with 50 rpm shaking. The antibody was precipitated with protein A-Sepharose (GE Healthcare, Chicago, IL, USA), and an in-gel kinase assay was carried out [30] with non-immunodepleted total protein.

### 2.7. Statistical Analysis

All experiments were independently conducted at least three times, and the data were analyzed using a *t*-test. Asterisks denote significant differences (** *p* < 0.01).

## 3. Results

### 3.1. The Gene Expression of GmRbohI1 Is Regulated by GmMPK6

**Figure 1:**
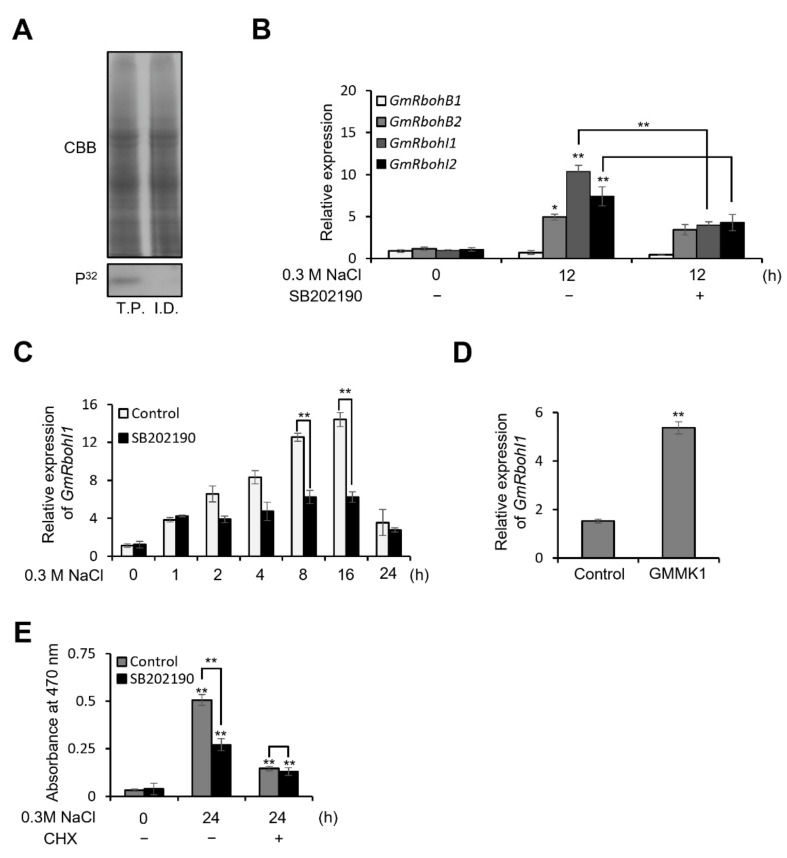
GmMPK6 increases expression of *GmRbohI1*. (**A**) Immunodepletion and in-gel kinase assay of GmMPK6. Seven-day-old soybean seedlings were treated with 0.3 M NaCl for 5 min, and total protein was isolated from the seedlings. The protein was incubated with anti-GmMPK6 and incubated for 3 h at 4 °C. Then, protein A-Sepharose was added and incubated for 3 h at 4 °C. After removing the beads, total protein was separated with SDS-PAGE and an in-gel kinase assay was carried out. T.P., total protein; I.D., GmMPK6-immunodepleted total protein. (**B**) Gene expression analysis of *GmRbohB1*, *GmRbohB2*, *GmRbohI1*, and *GmRbohI2* in salt treatment with MPK6 inhibitor pretreatment. Seven-day-old soybean seedlings were treated with 0.3 M NaCl for 12 h with or without 1 h pretreatment of 30 µM SB202190. Total RNA was isolated from the seedlings and RT-qPCR was carried out. *GmActin* was used as expression control. (**C**) The effect of SB202190 on the expression of *GmRbohI1* in salt-treated soybean seedlings. Seven-day-old soybean seedlings were treated with or without 30 μM SB202190 for 1 h followed by treatment with 0.3 M NaCl at designated time points. (**D**) Expression of *GmRbohI1* in GMMK1-expressing protoplasts. GMKK1 was transfected to soybean protoplast, and after 24 h, *GmRboh1* expression level was investigated. (**E**) The effect of CHX on ROS generation in salt-stressed soybean. Seven-day-old seedlings were treated with or without 200 μM CHX, and with or without 30 μM SB202190 for 1 h followed by treatment with 0.3 M NaCl and 0.5 mM XTT for 24 h with a designated combination. ROS generation was measured with a spectrophotometer at 470 nm. Data are means ± SD of three repeats: **, *p* < 0.01; *, *p* < 0.05.

It has been shown that a soybean MPK, GmMPK6, is activated during salt stress, and the MPK inhibitor SB202190 inhibits GmMPK6 activity and salt-induced ROS generation in salt stress [28]. Based on this observation, we hypothesized that GmMPK6 mediates the signaling pathway of ROS production under salt stress. To prove this, we first confirmed whether GmMPK6 is solely activated under salt-stress conditions with immunodepletion assay, and it showed that there is no remaining MPK activity when GmMPK6 is depleted (Figure 1A). 

It was previously shown that soybean Rboh enables the accumulation of ROS to amplify the initial salt stress signal in soybean roots under salt stress [19], and 17 *GmRboh* genes were identified in soybean [36]. As a first step to identify the *Rboh* genes that respond to salt stress, we investigated the Soybean Expression Atlas (https://venanciogroup.uenf.br/cgi-bin/gmax_atlas/search_gene_list.cgi (accessed on 21 August 2022) [37] to analyze expression profiles for 17 *GmRboh* genes under salt treatment. The results show that these *GmRboh* genes had different expression responses under salt conditions (Figure S2). The expression levels of *GmRbohB* and *GmRbohI* genes from clade I were similar, and *GmRbohI1* (Glyma.10G152200.1) showed the highest transcriptional induction by salt stress. To confirm the salt-induced gene expression experimentally and identify whether GmMPK6 can regulate the transcription of *GmRhoh* genes during salt stress, we applied RT-qPCR and investigated the expression of four representative salt-responsive *GmRboh* genes (*GmRbohB1*, *GmRbohB2*, *GmRbohI1*, and *GmRbohI2*) under 0.3 M NaCl treatment with a pretreatment of SB202190. *GmRbohB2*, *GmRbohI1*, and *GmRbohI2* showed significant induction by NaCl treatment, but the expression of *GmRbohI1* was significantly compromised by SB202190 pretreatment (Figure 1B). Therefore, we chose *GmRbohI1* as the candidate gene under downstream regulation of GmMPK6. 

To dissect the regulation of *GmRbohI1* expression by GmMPK6, we treated 0.3 M NaCl to seven-day-old soybean seedlings with or without SB202190 pretreatment and analyzed the gene expression of *GmRbohI1* in a time dependent manner. The transcription level of *GmRbohI1* was increased by NaCl treatment, but it was significantly reduced by SB202190 (Figure 1C). To further analyze the regulation, we examined the transcription level of *GmRbohI1* in soybean protoplasts expressing soybean MEK, GMKK1 (Glyma.07G003200.1), which is a direct activator of GmMPK6 (Figure S3). As a result, the expression of *GmRbohI1* was increased in GMKK1-expressing protoplasts compared to the control (Figure 1D). Additionally, cycloheximide (CHX), a de novo protein synthesis inhibitor, significantly reduced NaCl dependently, increased ROS generation, and abolished the ROS-reducing effect of SB202190 pretreatment as well (Figure 1E), indicating that SB202190-mediated inhibition of ROS generation was significantly inhibited by CHX. In other words, this means that GmMPK6 does not regulate GmRbohI1 activity; instead, GmMPK6 induces ROS generation by modulating the transcription level and further synthesis of *GmRbohI1* under salt-stress conditions. 

### 3.2. GmMPK6 Increases Salt Tolerance

**Figure 2:**
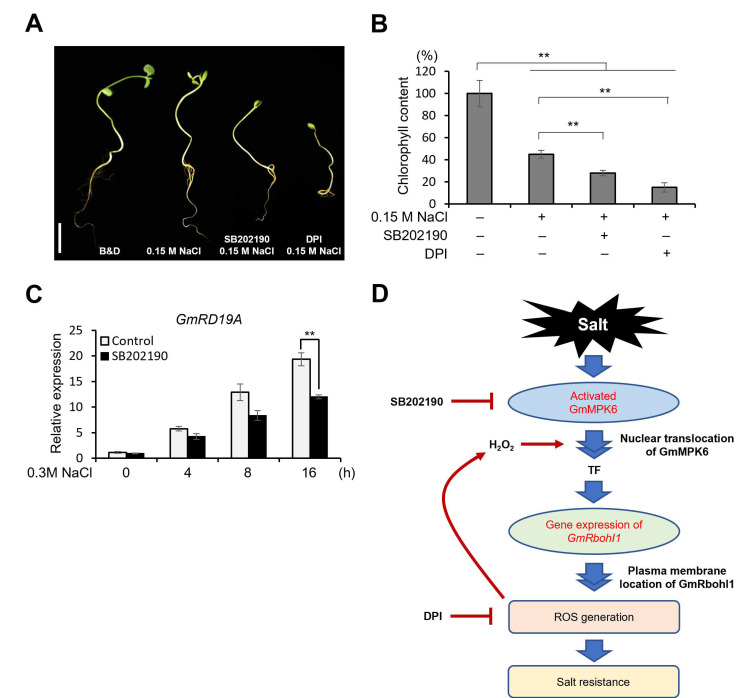
GmMPK6 increases salt tolerance. (**A**) The phenotype of NaCl with MPK inhibitor or NADPH-oxidase inhibitor-treated soybean seedling. Four-day-old seedlings were treated with 0.15 M NaCl and with or without 30 μM SB202190 or 50 μM DPI for 3 days. B & D solution-treated soybean seedlings were used as a control. Bar = 2 cm. (**B**) The chlorophyll contents of 0.15 M NaCl and inhibitors, respectively, co-treated soybean leaves. Ten-day-old soybean seedlings were treated with 0.15 M NaCl, 30 μM SB202190 + 0.15 M NaCl, and 50 μM DPI + 0.15 M NaCl for 5 days, respectively, and then chlorophyll contents were measured for leaves. B & D-solution-treated soybean seedlings were used as a control. (**C**) The effect of SB202190 on the expression of *GmRD19A* in NaCl-treated soybean seedlings. Seven-day-old seedlings were treated with 30 μM SB202190 for 1 h, followed by the treatment of 0.3 M NaCl for designated time points. For RT-qPCR, total RNA was isolated, and the amplification was carried out with gene-specific primers (Table S1). (**D**) Schematic diagram of GmMPK6-dependent ROS generation through the induction of *GmRbohI1*. Data are means ± SD of three repeats: **, *p* < 0.01.

Because the role of GmMPK6 in the salt tolerance of soybean has not yet been experimentally demonstrated, we first examined the phenotype of soybean seedlings with or without MPK inhibitor in salinity conditions. An NADPH-oxidase inhibitor, Diphenyleneiodonium chloride (DPI), reduces ROS generation in salt stress (Figure S4). We used DPI as a positive control because ROS plays important functions for salt tolerance in soybean [36]. The four-day-old soybean seedlings were treated with 0.15 M NaCl for 3 days with or without SB202190 or DPI co-treatment. The seedling development was delayed by 0.15 M NaCl, and the shoot did not develop in SB202190 co-treated seedlings, and even cotyledon did not open in DPI co-treated seedlings (Figure 2A). Secondly, we investigated phenotypic differences in leaf color and chlorophyll content using 10-day-old soybean seedlings. They were treated with 0.15 M NaCl with or without SB202190 or DPI co-treatment for 5 days. Under salinity conditions, the soybean leaves were wrinkled and turned slightly yellow. The SB202190 co-treated soybean leaves were more yellowish, and DPI-co-treated soybean leaves turned entirely yellow (Figure S5). The chlorophyll content was also significantly reduced in leaves with NaCl treatment, and more significantly reduced in SB202190 or DPI co-treated soybean leaves (Figure 2B). Thirdly, we analyzed the gene expressions of *GmRD19A* (Glyma.11G113500.1). The *GmRD19A* is a soybean homolog of *RD19A,* which is known as an essential gene for salt tolerance in Arabidopsis [38] and showed a time-dependent transcriptional induction by NaCl or H_2_O_2_ treatment (Figure S6). As shown in Figure 2C, the salt-dependent induction of *GmRD19A* was significantly inhibited by SB202190 pretreatment. This means that GmMPK6-ROS generation is responsible for the induction of *GmRD19A* under salt-stress conditions. Taken together, we experimentally demonstrated that GmMPK6-mediated ROS generation positively regulates salt tolerance in soybean.

These findings are schematically represented in Figure 2D. GmMPK6 is activated by salt stress and translocated to a nucleus by H_2_O_2_ to activate the gene expression of *GmRbohI1*. The produced GmRbohI1 increases ROS generation, and ultimately leads to salt-stress-tolerance phenotypes, including relieving seedling growth inhibition and chlorophyll degradation and the induction of defense genes such as *GmRD19A*. 

## 4. Discussion

Our previous studies revealed that GmMPK6 was activated and increased ROS generation under salt-stress conditions [27,28]; however, the regulation mechanism of how GmMPK6 increases ROS generation has not yet been elucidated. As Rboh was known to generate ROS in response to salt stress in several plant species [16,17,18,19,20,21], we investigated the correlation between GmMPK6 activity and Rboh expression in soybean under salt stress. *GmRbohI1*, a homolog of Arabidopsis *RbohD*, exhibited the highest increase in expression among 17 *Rboh* genes of soybean under salt-stress conditions (Figure S2). The *GmRbohI1* transcriptional induction effect by salt treatment was reduced by SB202190, an inhibitor of MPK (Figure 1B,C). GMKK1, an activator of GmMPK6, increased the expression of *GmRbohI1* in soybean protoplasts (Figure 1D), and SB202190-dependent ROS reduction was not detected in the presence of CHX (Figure 1E). These results suggest that GmRbohI1 is under the regulation of GmMPK6 in salt-stress conditions, and the regulation is at the transcriptional level rather than the post-translational level. GmMPK6 mediated induction of *GmRbohI1* increased ROS level, and the ROS increased the salt-tolerance accordingly (Figure 1 and Figure 2). This process is similarly conserved in *Arabidopsis thaliana*, where it is known that activated MPK3 and MPK6 induce the expression of the *RbohD* gene in the biological defense response to Vd-toxins [39]. However, as only GmMPK6 was found to be activated in soybean under salt-stress conditions, it is presumed that the functions of MPK orthologs in response to external stimuli differentially evolved depending on the plant species.

Under salt stress, Arabidopsis MPK6 is activated by phosphatidic acid (PA) and activates SOS1 with phosphorylation [40]. Further, Phospholipase α1 increases NADPH-oxidase activity via PA [41]. This suggests the possibility that GmMPK6 mediates salt-induced GmRboh activation, as GmMPK6 is also activated by PA early in salt stress [27]. However, there are two reasons to infer that this process may be an indirect process involving other factors between GmMPK6 and GmRboh. First, GmMPK6 is translocated to the nucleus within one hour of the salt stress [29]. Because Rboh normally acts at the plasma membrane, the spatial separation of GmMPK6 and Rboh reduces the chance of direct interaction. Second, the activation of *Rboh* by MPK in tobacco and Arabidopsis is known to be mediated by transcription factors. In *Nicotiana benthamiana*, MAPK signaling mediated by NbMKK2 regulates cis elements of the *NbRbohB* promoter by phosphorylating WRKY transcription factors (e.g., WRKY7/WRKY8/WRKY9/WRKY11) during effector-triggered ROS bursts [42]. Arabidopsis plants overexpressing the ethylene response transcription factor *ERF104* increased *RbohD* expression 3.7-fold, suggesting that *RbohD* is a possible target of ERF104 [43]. However, although AtMPK6-*ERF104* has been demonstrated to regulate stress response genes via the GCC box in Arabidopsis, it does not appear to directly regulate *AtRbohD* expression via the GCC box as no GCC box was found in the 2500 bp region upstream of *AtRbohD* [44]. Elucidating the signaling components between GmMPK6 and *GmRboh* expression under salt stress conditions in soybean will be the major focus of future studies.

ROS is generated and MPK is activated in salinity condition, and they affect each other’s activity and generation [22,28,39,45]. However, it has not been studied which one comes first. GmMPK6 is activated by GmRboh dependently-generated ROS after 10 min of salt treatment [28] and translocates to the nucleus after one hour of the treatment [29]. SB202190 pretreatment reduced ROS generation after four hours of the treatment, meaning that ROS activates GmMPK6 first and GmMPK6 increases ROS generation later.

In addition, we investigated whether the GmMPK6–GmRbohI1 module is involved in enhancing the salt tolerance of soybean. When seedlings were treated with MPK inhibitor, SB202190, or NADPH-oxidase inhibitor, DPI, the salt stress tolerance phenotype was significantly repressed in various aspects, including the growth inhibition of seedlings, leaf chlorophyll content, and expression of *GmRD19A* (Figure 2A–C and Figure S5). Based on these observations, we conclude that the GmMPK6–GmRbohI1 module is positively involved in the salt tolerance of soybean. It was reported that the GmSIN1 transcription factor, transcriptionally induced by various environmental stresses, including salt stress in soybeans, directly binds to the promoter region of *GmRbohI1* and increases ROS production. This positive feed-forward system is known to contribute to the increased salt tolerance of soybean roots [19]. 

Rboh transfer electrons to generate O_2_^−^, and it O_2_^−^ is converted to H_2_O_2_ by superoxide dismutase [46]. H_2_O_2_ is a central stress signaling regulator, but it should regulate the concentration for non-toxic levels. Therefore, the precise regulation of ROS levels is required for ROS homeostasis to prevent detrimental effects due to excessive ROS accumulation [47]. GmSALT3, a protein belonging to the plant cation/proton exchanger family, was recently shown to scavenge ROS under salt-stress conditions [48]. For an in-depth understanding of the salt tolerance mechanism of soybean, further biochemical and genetic studies will be needed to investigate the regulatory components linking the GmMPK6 and GmSIN1 transcription factors and to identify the crosstalk among the GmMPK6 signaling module, GmRboh ROS generator, and GmSALT3 ROS scavenger.

## Data Availability

The data presented in this study are available in the article or in the Supplementary Materials.

## Supplementary Materials

The following supporting information can be downloaded at: https://www.mdpi.com/article/10.3390/antiox12030601/s1, Figure S1. Gene expression of *GmRbohI1* with three different reference genes. Figure S2: Expression profiles of *GmRboh* genes under the salt condition; Figure S3: In vitro kinase assay of GMKK1 with GmMPKs; Figure S4: ROS generation in soybean seedings by 0.3 M NaCl treatment with or without pretreatment of SB202190 or DPI; Figure S5: Phenotype of soybean leaves co-treated with 150 mM NaCl and various inhibitors; Figure S6: Expression of *GmRD19A* in NaCl- or H_2_O_2_-treated soybean seedlings; Table S1: List of primers in this study.
