# Supplementary material for: GmMPK6 Positively Regulates Salt Tolerance through Induction of GmRbohI1 in Soybean
Source: Antioxidants (Basel). 2023 Feb 28;12(3):601. doi: 10.3390/antiox12030601 (PMC10045776; doi:10.3390/antiox12030601)
Supplement: Supplementary file 1 [file antioxidants-12-00601-s001.zip › antioxidants-2174211-supplementary.pptx]

## Slide 1
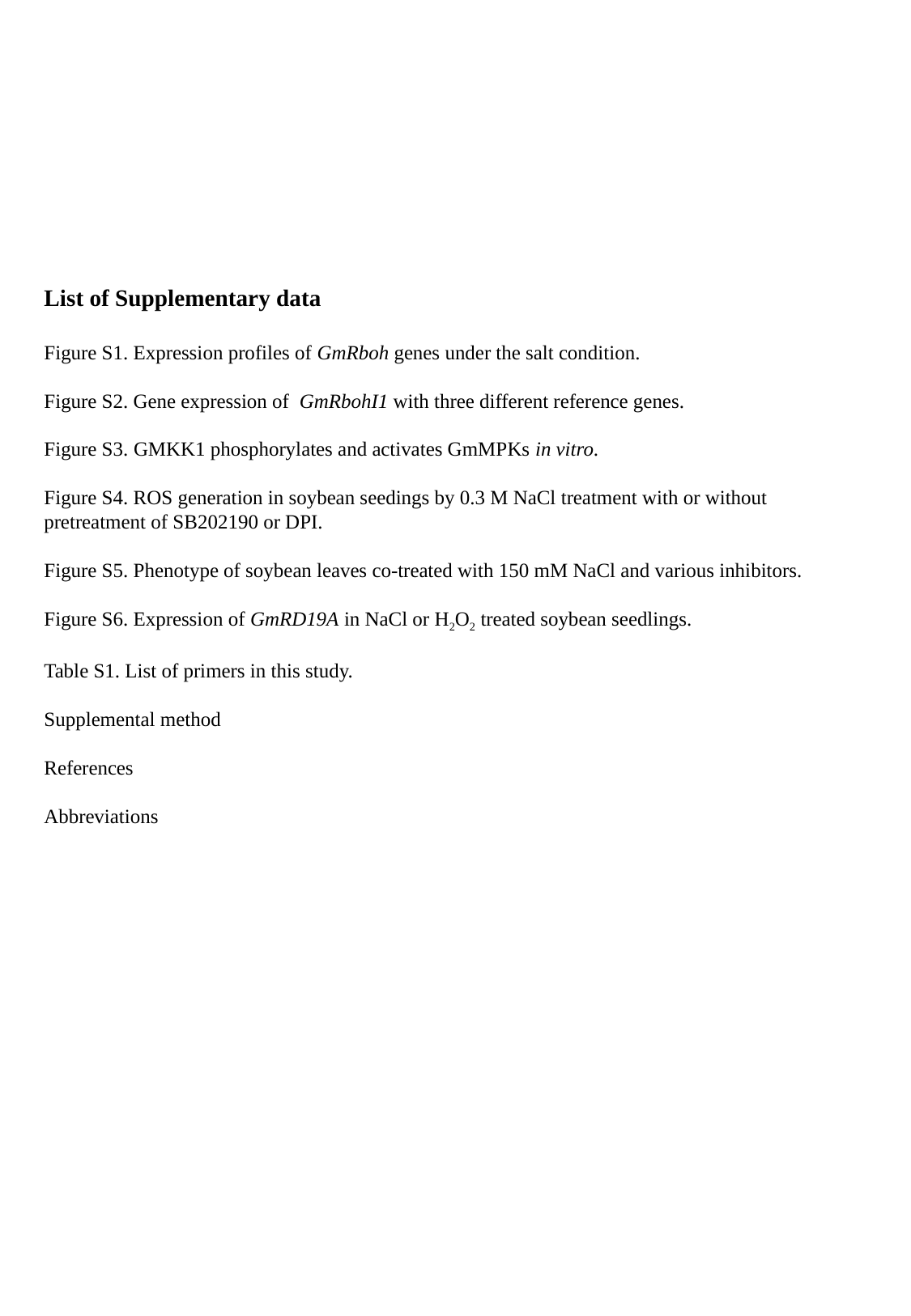

List of Supplementary data
Figure S1. Expression profiles of GmRboh genes under the salt condition.
Figure S2. Gene expression of GmRbohI1 with three different reference genes.
Figure S3. GMKK1 phosphorylates and activates GmMPKs in vitro.
Figure S4. ROS generation in soybean seedings by 0.3 M NaCl treatment with or without pretreatment of SB202190 or DPI.
Figure S5. Phenotype of soybean leaves co-treated with 150 mM NaCl and various inhibitors.
Figure S6. Expression of GmRD19A in NaCl or H2O2 treated soybean seedlings.
Table S1. List of primers in this study.
Supplemental method
References
Abbreviations

## Slide 2
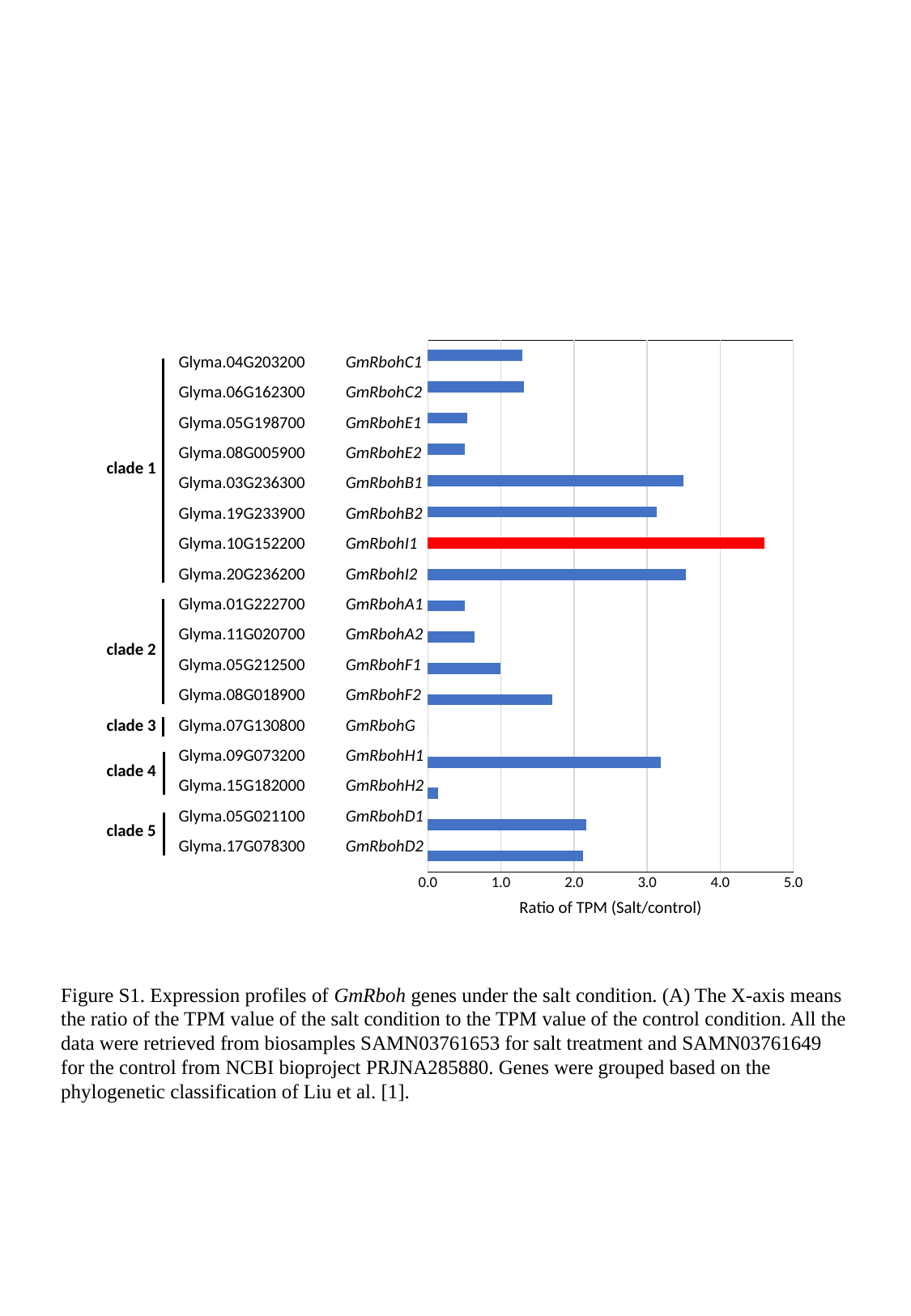

### Chart
| Category | Ratio |
|---|---|
| GmRbohD2 | 2.125558404457891 |
| GmRbohD1 | 2.165738086578432 |
| GmRbohH2 | 0.1358124058323804 |
| GmRbohH1 | 3.183133903133903 |
| GmRbohG | 0.0 |
| GmRbohF2 | 1.7004715089669666 |
| GmRbohF1 | 0.9886855153967404 |
| GmRbohA2 | 0.6363297706323496 |
| GmRbohA1 | 0.5043437821835954 |
| GmRbohI2 | 3.5339214309197935 |
| GmRbohI1 | 4.603736405865669 |
| GmRbohB2 | 3.133508108913383 |
| GmRbohB1 | 3.4953512402369458 |
| GmRbohE2 | 0.5009212966471508 |
| GmRbohE1 | 0.537154991210332 |
| GmRbohC2 | 1.3100814693487104 |
| GmRbohC1 | 1.2962999523875434 || clade 1 | Glyma.04G203200 | GmRbohC1 |
| --- | --- | --- |
| | Glyma.06G162300 | GmRbohC2 |
| | Glyma.05G198700 | GmRbohE1 |
| | Glyma.08G005900 | GmRbohE2 |
| | Glyma.03G236300 | GmRbohB1 |
| | Glyma.19G233900 | GmRbohB2 |
| | Glyma.10G152200 | GmRbohI1 |
| | Glyma.20G236200 | GmRbohI2 |
| clade 2 | Glyma.01G222700 | GmRbohA1 |
| | Glyma.11G020700 | GmRbohA2 |
| | Glyma.05G212500 | GmRbohF1 |
| | Glyma.08G018900 | GmRbohF2 |
| clade 3 | Glyma.07G130800 | GmRbohG |
| clade 4 | Glyma.09G073200 | GmRbohH1 |
| | Glyma.15G182000 | GmRbohH2 |
| clade 5 | Glyma.05G021100 | GmRbohD1 |
| | Glyma.17G078300 | GmRbohD2 |
Ratio of TPM (Salt/control)
Figure S1. Expression profiles of GmRboh genes under the salt condition. (A) The X-axis means the ratio of the TPM value of the salt condition to the TPM value of the control condition. All the data were retrieved from biosamples SAMN03761653 for salt treatment and SAMN03761649 for the control from NCBI bioproject PRJNA285880. Genes were grouped based on the phylogenetic classification of Liu et al. [1].

## Slide 3
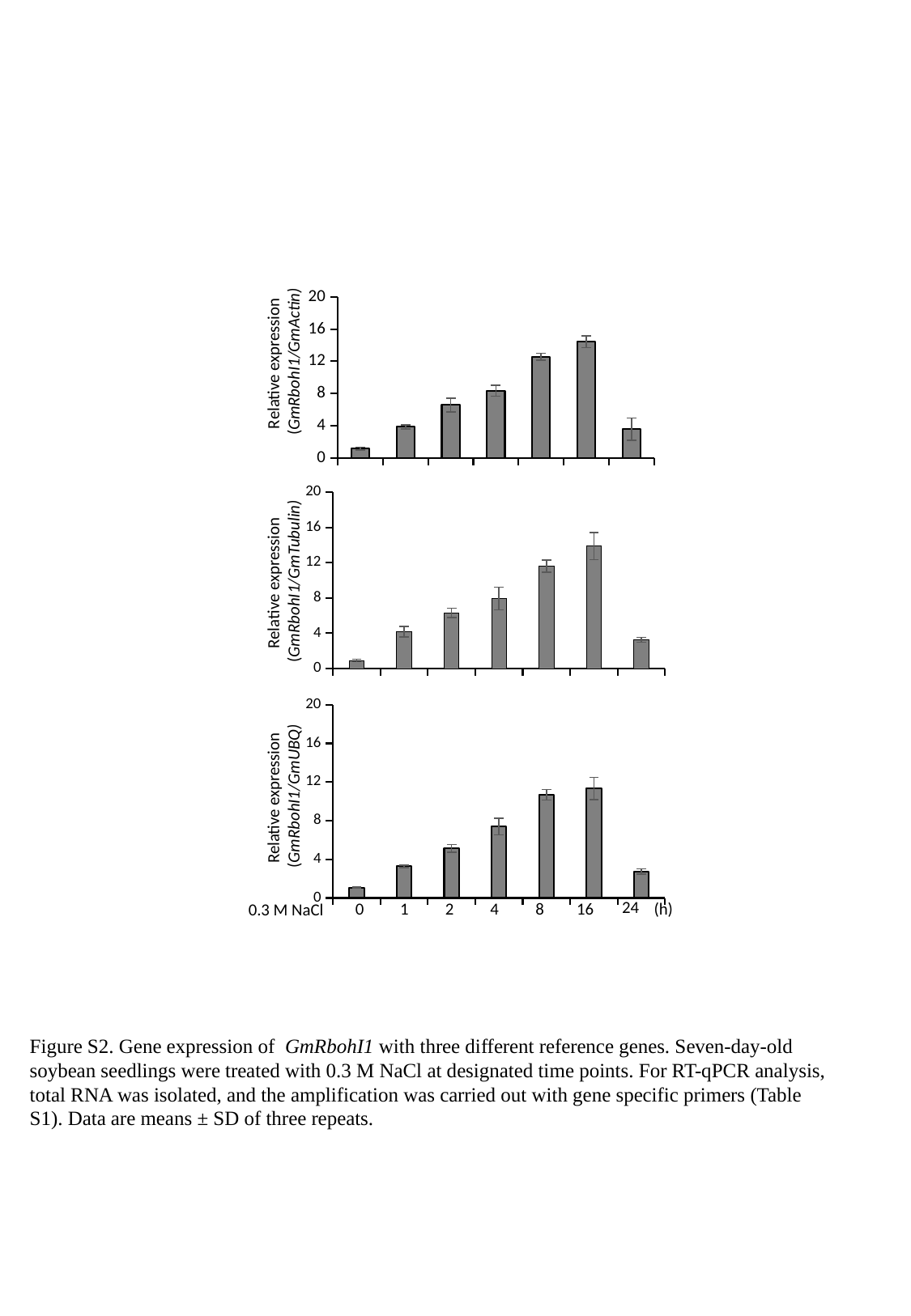

### Chart
| Category | Control |
|---|---|
| | 1.14 |Relative expression
 (GmRbohI1/GmActin)
### Chart
| Category | |
|---|---|
| | 0.92 |Relative expression
 (GmRbohI1/GmTubulin)
### Chart
| Category | |
|---|---|
| | 1.07 |Relative expression
 (GmRbohI1/GmUBQ)
24
(h)
8
16
1
4
0
2
0.3 M NaCl
Figure S2. Gene expression of GmRbohI1 with three different reference genes. Seven-day-old soybean seedlings were treated with 0.3 M NaCl at designated time points. For RT-qPCR analysis, total RNA was isolated, and the amplification was carried out with gene specific primers (Table S1). Data are means ± SD of three repeats.

## Slide 4
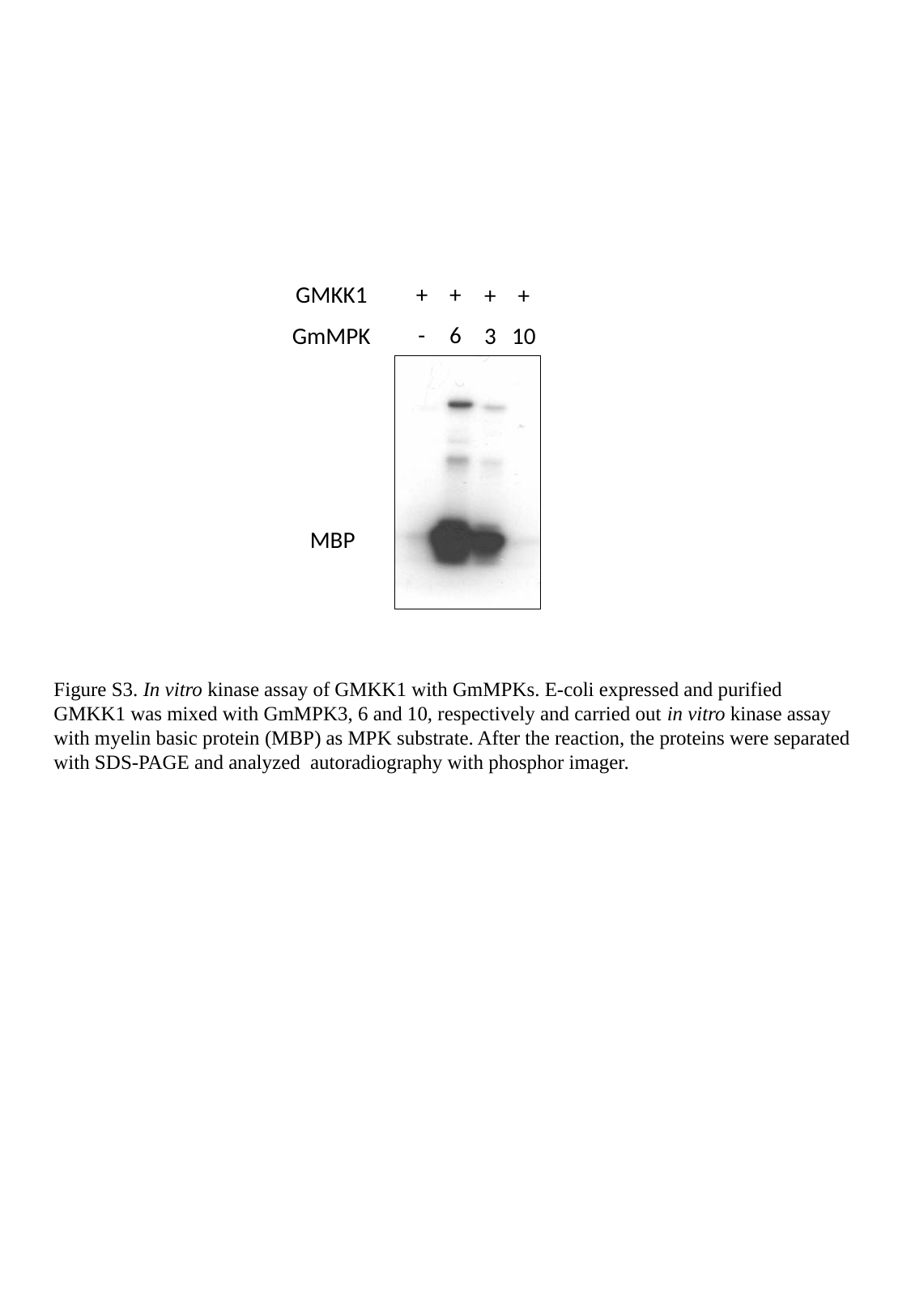

+
GMKK1
+
+
+
6
-
3
10
GmMPK
MBP
Figure S3. In vitro kinase assay of GMKK1 with GmMPKs. E-coli expressed and purified GMKK1 was mixed with GmMPK3, 6 and 10, respectively and carried out in vitro kinase assay with myelin basic protein (MBP) as MPK substrate. After the reaction, the proteins were separated with SDS-PAGE and analyzed autoradiography with phosphor imager.

## Slide 5
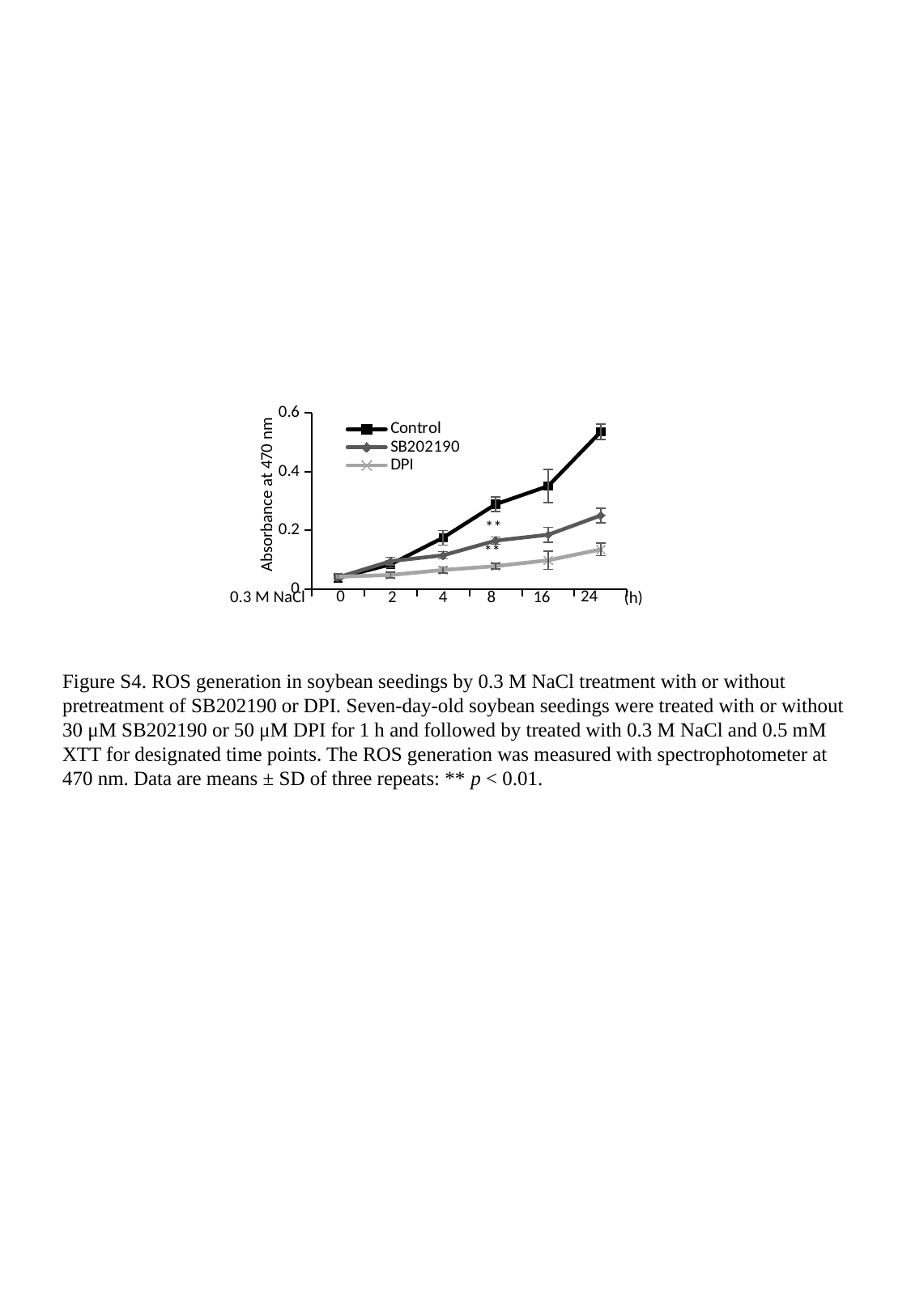

### Chart
| Category | Control | SB202190 | DPI |
|---|---|---|---|
| | 0.038 | 0.04 | 0.042 |Absorbance at 470 nm
**
**
24
0
16
2
8
4
0.3 M NaCl
(h)
Figure S4. ROS generation in soybean seedings by 0.3 M NaCl treatment with or without pretreatment of SB202190 or DPI. Seven-day-old soybean seedings were treated with or without 30 μM SB202190 or 50 μM DPI for 1 h and followed by treated with 0.3 M NaCl and 0.5 mM XTT for designated time points. The ROS generation was measured with spectrophotometer at 470 nm. Data are means ± SD of three repeats: ** p < 0.01.

## Slide 6
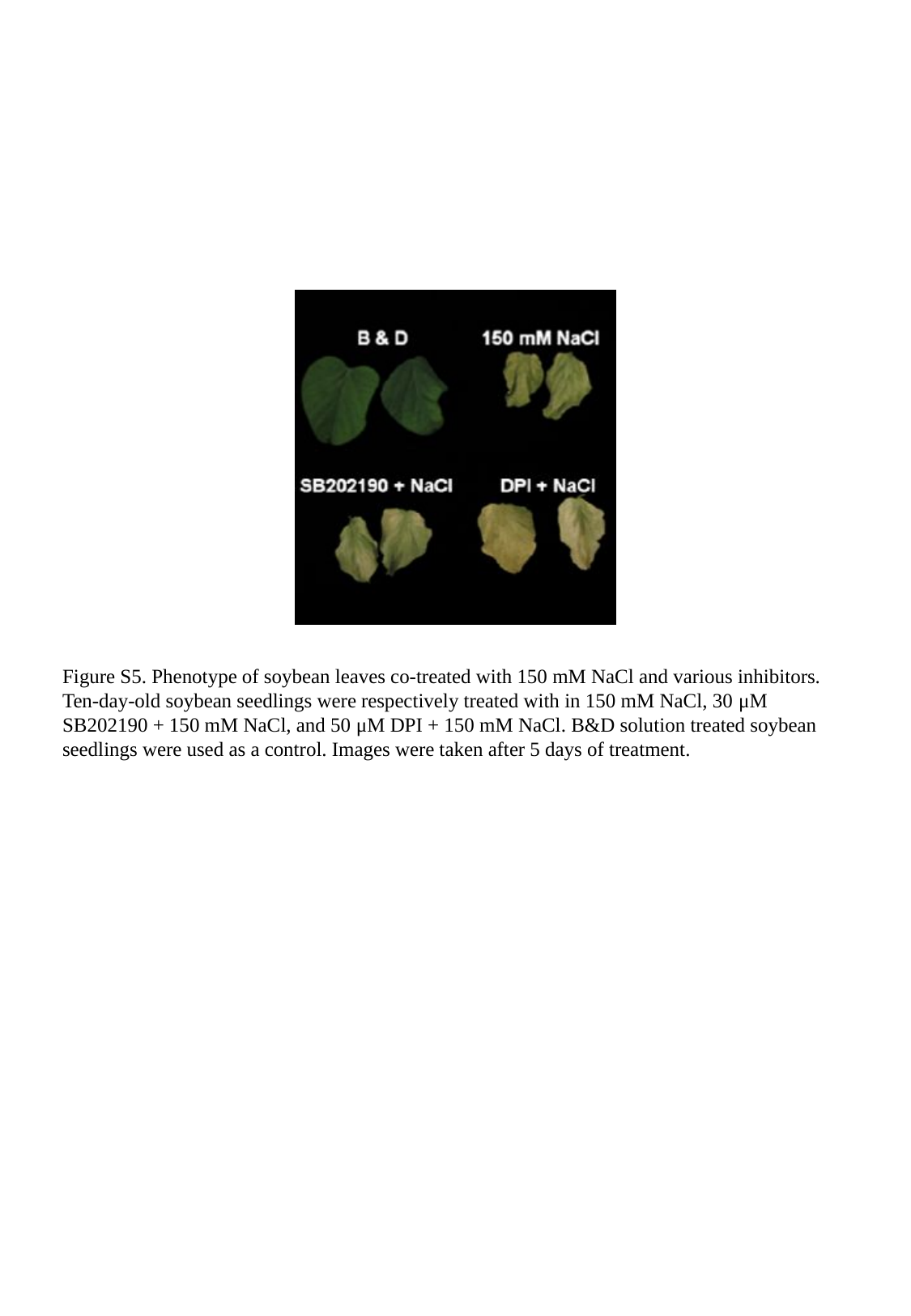

Figure S5. Phenotype of soybean leaves co-treated with 150 mM NaCl and various inhibitors. Ten-day-old soybean seedlings were respectively treated with in 150 mM NaCl, 30 μM SB202190 + 150 mM NaCl, and 50 μM DPI + 150 mM NaCl. B&D solution treated soybean seedlings were used as a control. Images were taken after 5 days of treatment.

## Slide 7
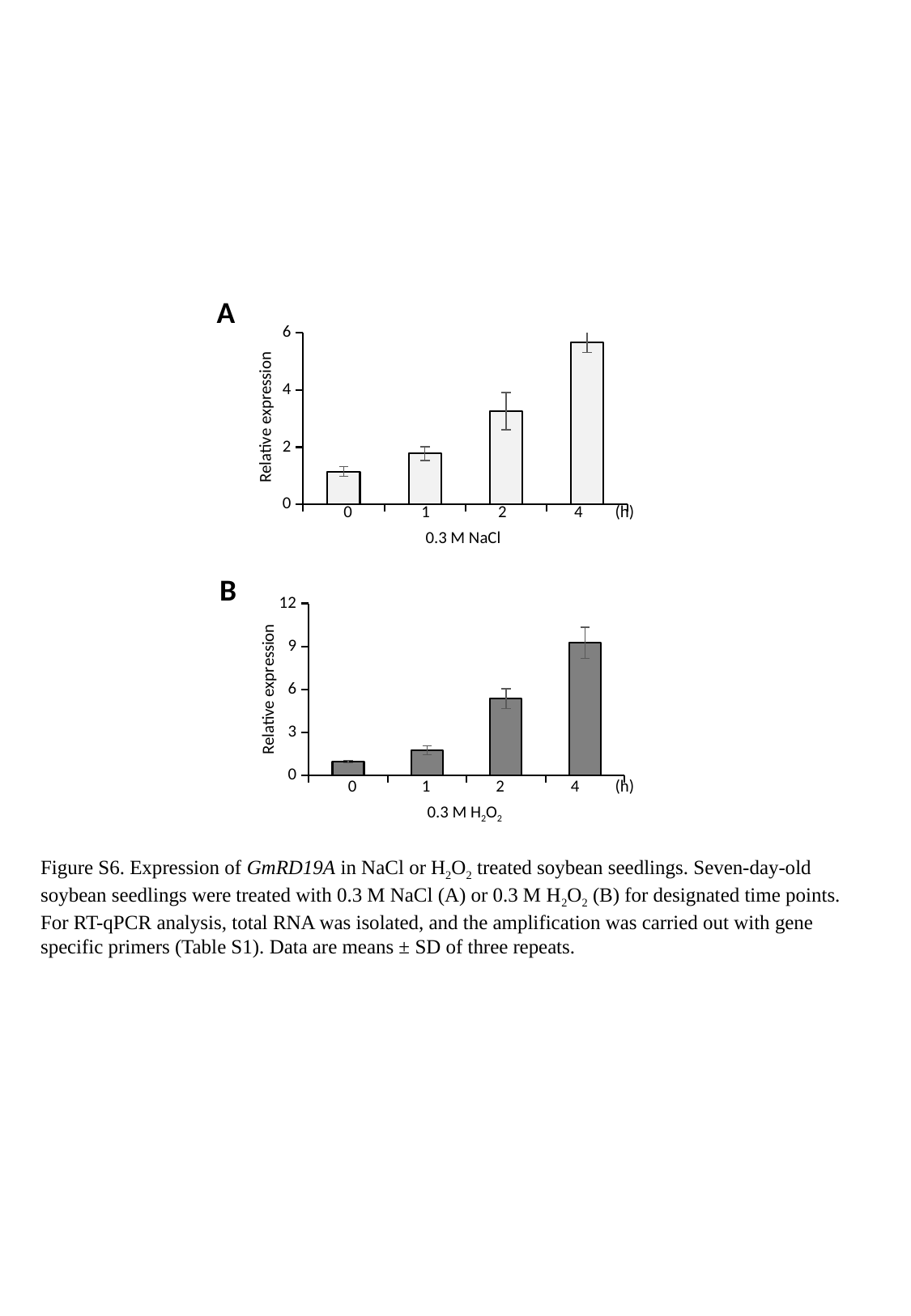

A
### Chart
| Category | Control |
|---|---|
| | 1.15 |Relative expression
0
(h)
1
4
2
0.3 M NaCl
B
### Chart
| Category | Control |
|---|---|
| | 0.97 |Relative expression
(h)
4
2
1
0
0.3 M H2O2
Figure S6. Expression of GmRD19A in NaCl or H2O2 treated soybean seedlings. Seven-day-old soybean seedlings were treated with 0.3 M NaCl (A) or 0.3 M H2O2 (B) for designated time points. For RT-qPCR analysis, total RNA was isolated, and the amplification was carried out with gene specific primers (Table S1). Data are means ± SD of three repeats.

## Slide 8
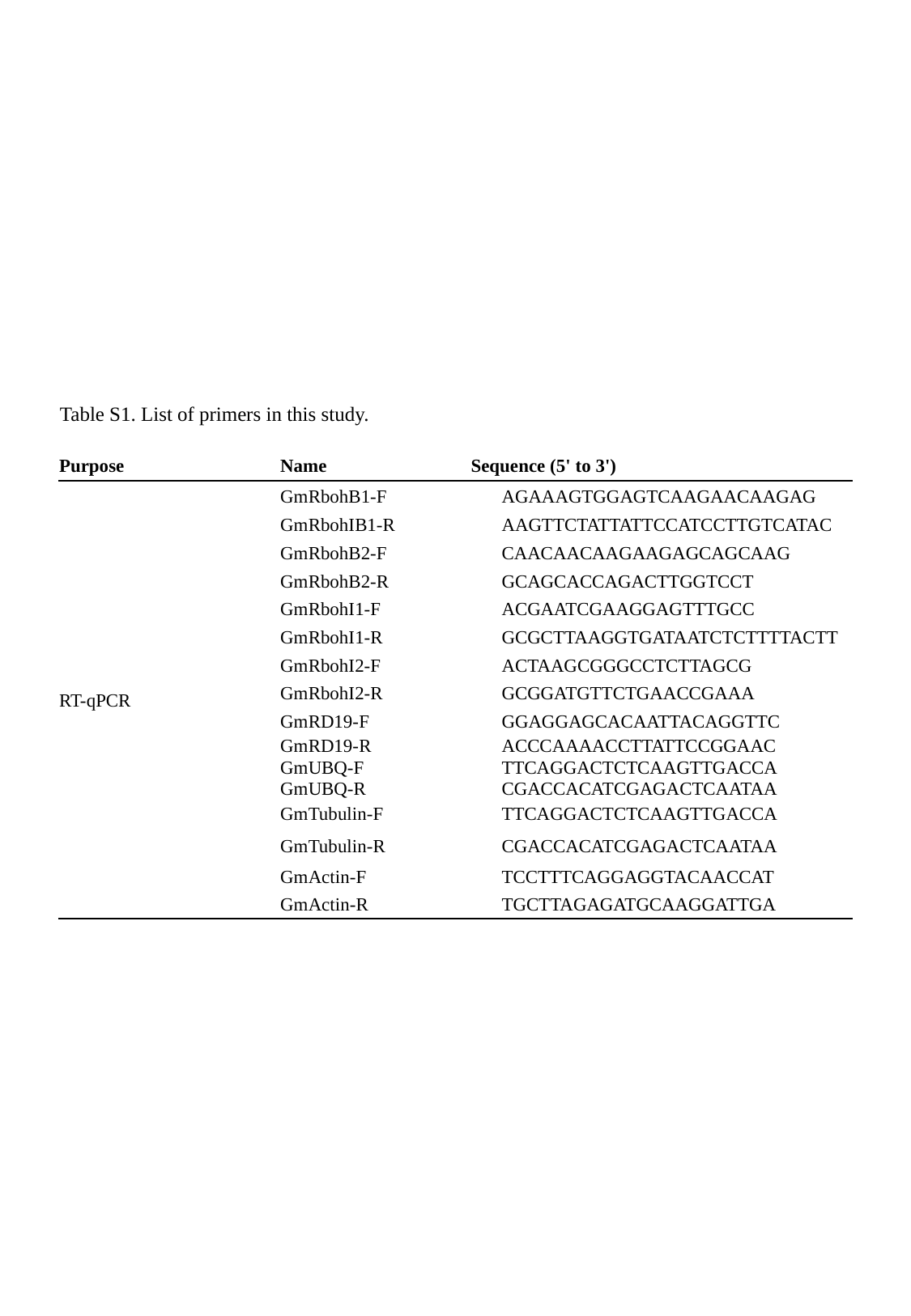

Table S1. List of primers in this study.
| Purpose | Name Sequence (5' to 3') | |
| --- | --- | --- |
| RT-qPCR | GmRbohB1-F | AGAAAGTGGAGTCAAGAACAAGAG |
| | GmRbohIB1-R | AAGTTCTATTATTCCATCCTTGTCATAC |
| | GmRbohB2-F | CAACAACAAGAAGAGCAGCAAG |
| | GmRbohB2-R | GCAGCACCAGACTTGGTCCT |
| | GmRbohI1-F | ACGAATCGAAGGAGTTTGCC |
| | GmRbohI1-R | GCGCTTAAGGTGATAATCTCTTTTACTT |
| | GmRbohI2-F | ACTAAGCGGGCCTCTTAGCG |
| | GmRbohI2-R | GCGGATGTTCTGAACCGAAA |
| | GmRD19-F | GGAGGAGCACAATTACAGGTTC |
| | GmRD19-R | ACCCAAAACCTTATTCCGGAAC |
| | GmUBQ-F | TTCAGGACTCTCAAGTTGACCA |
| | GmUBQ-R | CGACCACATCGAGACTCAATAA |
| | GmTubulin-F | TTCAGGACTCTCAAGTTGACCA |
| | GmTubulin-R | CGACCACATCGAGACTCAATAA |
| | GmActin-F | TCCTTTCAGGAGGTACAACCAT |
| | GmActin-R | TGCTTAGAGATGCAAGGATTGA |

## Slide 9
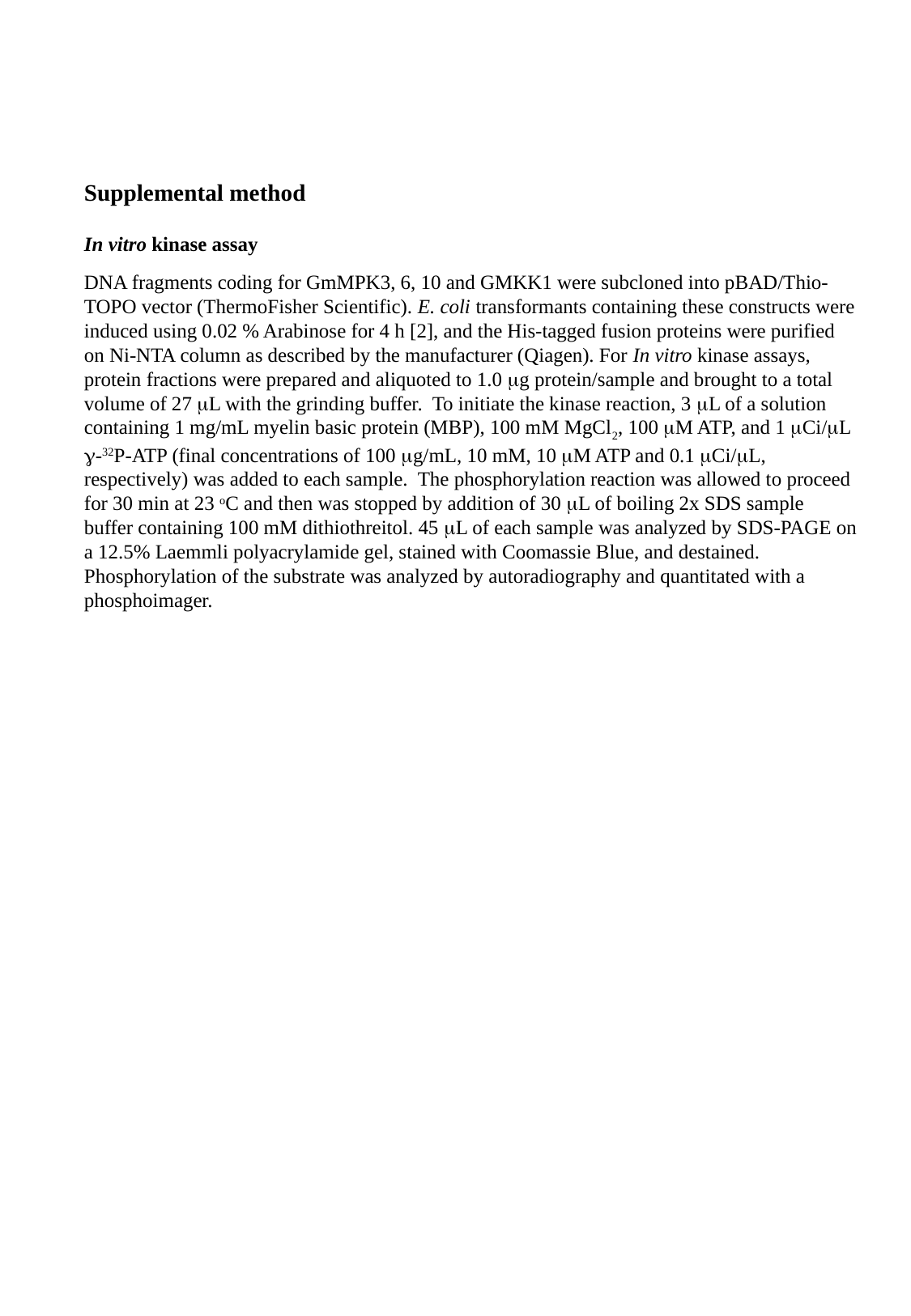

Supplemental method
In vitro kinase assay
DNA fragments coding for GmMPK3, 6, 10 and GMKK1 were subcloned into pBAD/Thio-TOPO vector (ThermoFisher Scientific). E. coli transformants containing these constructs were induced using 0.02 % Arabinose for 4 h [2], and the His-tagged fusion proteins were purified on Ni-NTA column as described by the manufacturer (Qiagen). For In vitro kinase assays, protein fractions were prepared and aliquoted to 1.0 μg protein/sample and brought to a total volume of 27 μL with the grinding buffer. To initiate the kinase reaction, 3 μL of a solution containing 1 mg/mL myelin basic protein (MBP), 100 mM MgCl2, 100 μM ATP, and 1 Ci/μL γ‑32P-ATP (final concentrations of 100 μg/mL, 10 mM, 10 μM ATP and 0.1 Ci/μL, respectively) was added to each sample. The phosphorylation reaction was allowed to proceed for 30 min at 23 oC and then was stopped by addition of 30 μL of boiling 2x SDS sample buffer containing 100 mM dithiothreitol. 45 μL of each sample was analyzed by SDS-PAGE on a 12.5% Laemmli polyacrylamide gel, stained with Coomassie Blue, and destained. Phosphorylation of the substrate was analyzed by autoradiography and quantitated with a phosphoimager.

## Slide 10
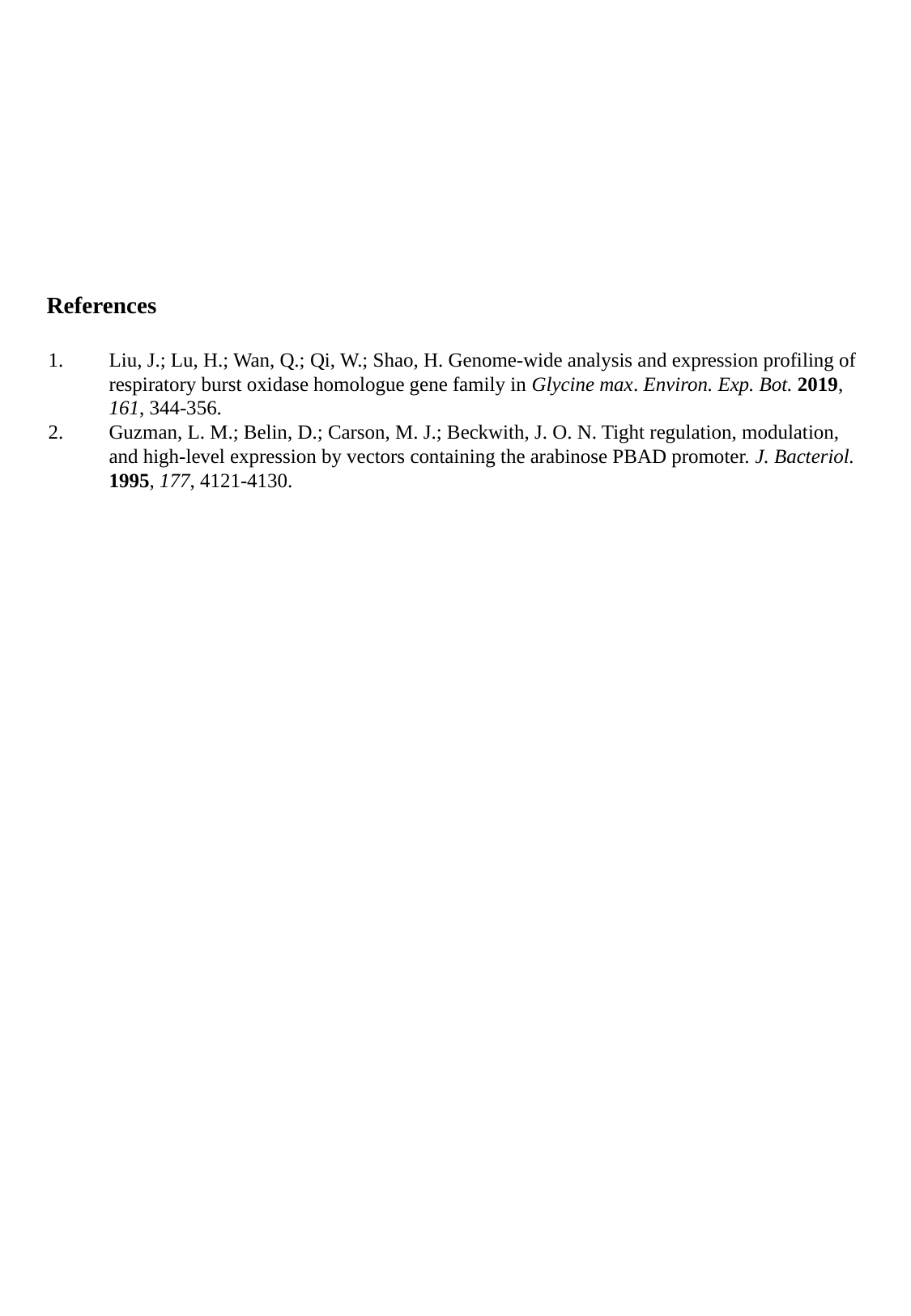

References
Liu, J.; Lu, H.; Wan, Q.; Qi, W.; Shao, H. Genome-wide analysis and expression profiling of respiratory burst oxidase homologue gene family in Glycine max. Environ. Exp. Bot. 2019, 161, 344-356.
Guzman, L. M.; Belin, D.; Carson, M. J.; Beckwith, J. O. N. Tight regulation, modulation, and high-level expression by vectors containing the arabinose PBAD promoter. J. Bacteriol. 1995, 177, 4121-4130.

## Slide 11
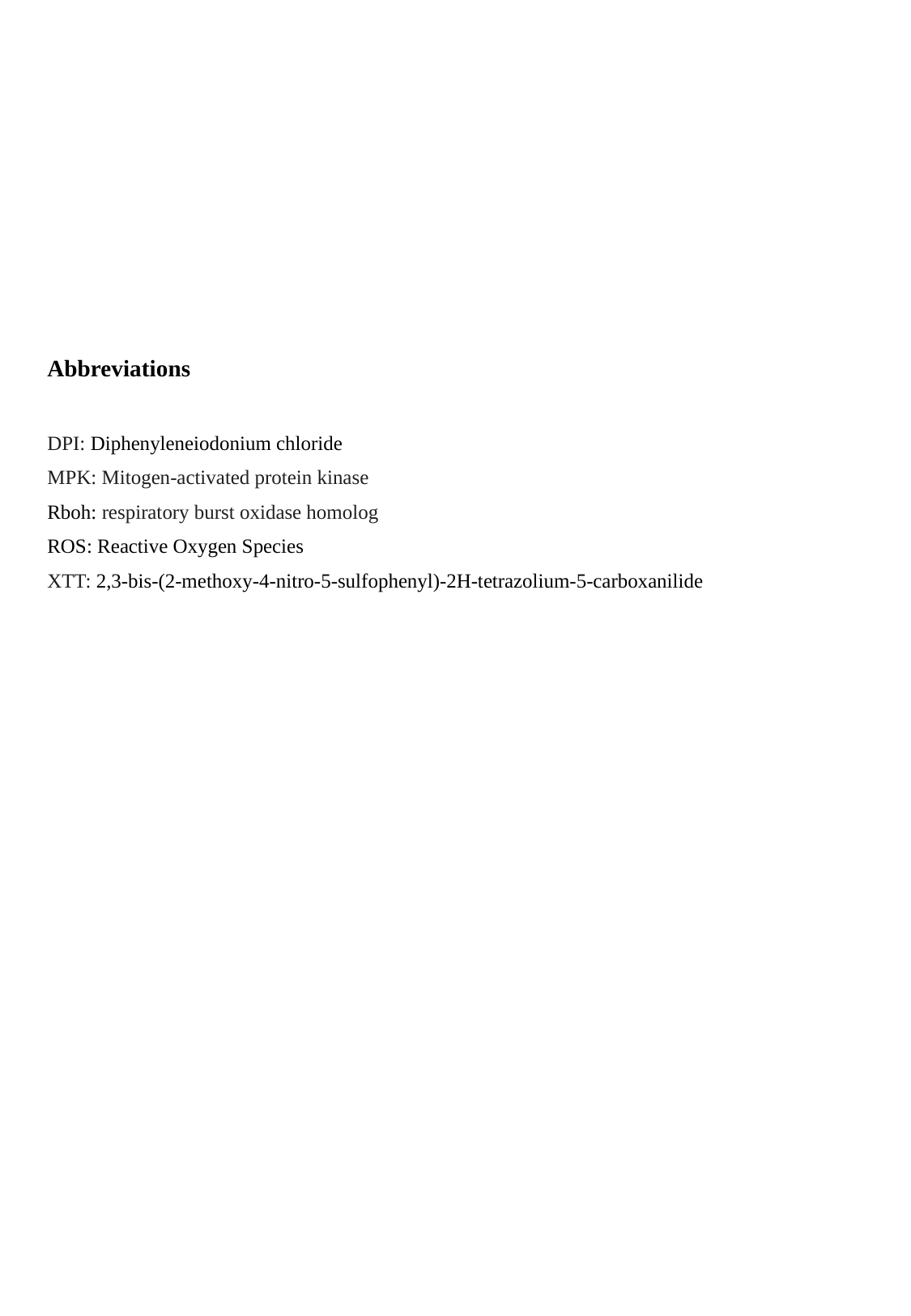

Abbreviations
DPI: Diphenyleneiodonium chloride
MPK: Mitogen-activated protein kinase
Rboh: respiratory burst oxidase homolog
ROS: Reactive Oxygen Species
XTT: 2,3-bis-(2-methoxy-4-nitro-5-sulfophenyl)-2H-tetrazolium-5-carboxanilide
